# Induction of Energy Expenditure by Sitagliptin Is Dependent on GLP-1 Receptor

**DOI:** 10.1371/journal.pone.0126177

**Published:** 2015-05-04

**Authors:** Felicia Goldsmith, Michael J. Keenan, Anne M. Raggio, Xin Ye, Zheng Hao, Holiday Durham, James Geaghan, Weiping Jia, Roy J. Martin, Jianping Ye

**Affiliations:** 1 Louisiana State University Agricultural Center, Baton Rouge, Louisiana 70803, United States of America; 2 Antioxidant and Gene Regulation Laboratory, Pennington Biomedical Research Center, Louisiana State University System, Baton Rouge, Louisiana 70808, United States of America; 3 Department of Endocrinology and Metabolism, Shanghai Jiao Tong University Affiliated Sixth People’s Hospital, Shanghai 200233, China; 4 Western Human Nutrition Research Center, Davis, California 95616, United States of America; 5 Department of Food Science and Technology, University of California Davis, Davis, California 95616, United States of America; University of Toronto, CANADA

## Abstract

Sitagliptin (SG) increases serum GLP-1 (Glucagon-like peptide-1) through inhibition of the hormone degradation. Resistant starch (RS) induces GLP-1 expression by stimulating L-cells in the intestine. Sitagliptin and resistant starch may have a synergistic interaction in the induction of GLP-1. This possibility was tested in current study in a mouse model of type 2 diabetes. Hyperglycemia was induced in the diet-induced obese mice by a signal injection of streptozotocin (STZ). Sitagliptin (0.4g/100g diet) was tested in the mice (n = 55) with dietary RS (HAM-RS2) at three dosages (0, 15, or 28g/100g diet). Energy and glucose metabolism were monitored in the evaluation of synergistic activity, and GLP-1 activity was determined in the GLP-1 receptor knockout (KO) mice. In the wild type mice, body weight and adiposity were reduced by sitagliptin, which was enhanced by RS (28g). Serum GLP-1 was induced and energy expenditure was enhanced by sitagliptin. Fasting glucose, insulin, and leptin levels were decreased by sitagliptin. The sitagliptin effects were lost in the KO mice (n = 25) although induction of serum GLP-1 by sitagliptin was even stronger in KO mice. The data suggests that sitagliptin is able to reduce adiposity and insulin resistance through induction of energy expenditure. The effect of sitagliptin is partially enhanced by RS. GLP-1 receptor may regulate serum GLP-1 by facilitating the hormone clearance.

## Introduction

The drug sitagliptin (SG), made by Merck & Co., LLC, was first marketed in the United States in 2006 [1] for improving glucose metabolism in patients with type 2 diabetes mellitus. The mechanism of sitagliptin action is up-regulation of serum glucagon-like peptide-1 (GLP-1) by inhibition of GLP-1 degradation. GLP-1 is produced by L-cells in small and large intestines in response to nutrient intake [2]. GLP-1 is degraded by dipeptidyl peptidase-4 (DPP-4) in the blood stream [2]. Sitagliptin is a DPP-4 inhibitor. Sitagliptin improves glucose metabolism and insulin sensitivity in both human and animal models without reducing body weight. In contrast, GLP-1 agonists reduce both blood glucose and body weight [3, 4].

Resistant starch (RS) is a type of dietary fiber that stimulates GLP-1 secretion in L-cells through fermentation in the large gut. Short chain fatty acids (SCFAs) are generated in the fermentation to stimulate L-cells for GLP-1 expression and secretion [5,6]. Supplementation with resistant type 2 (HAM-RS2) has been shown to reduce body fat. The mechanism is related to: (a) diluting the energy density of the diet, and (b) increasing β-oxidation of fatty acids [7,8]. Fermentation of dietary fiber is reduced by high-fat diet (HFD), resulting in attenuation of the beneficial effects resistant starch [9].

We hypothesize that sitagliptin and resistant starch may have a synergistic interaction in the induction of GLP-1. To test this possibility, we conducted two cohort studies in a mouse model of type 2 diabetes. In the first study, sitagliptin was tested with different doses of HAM-RS2 to determine the synergy. In the second study, the role of GLP-1 was tested in GLP-1 receptor KO mice.

## Methods

### Animal models

All study procedures were approved by the IACUC committee at the Pennington Biomedical Research Center. In the first study, fifty five (N = 55), 7-week-old male C57BL/6 (WT) mice were purchased from the JAX Laboratories (Bar Harbor, Maine), and housed individually in shoebox cages in a climate-controlled environment (21–22°C, 55% humidity) with a 12:12 hour light-dark cycle illuminated at 7 AM. The mice were fed a semi-purified HFD (42% energy) in order to induce weight gain. This pre-study diet also contained a small amount of HAM-RS2 (3% weight) in an attempt to maintain proper fermentative microbial communities despite dietary fat content. Mice were given one injection of a low dose of streptozotocin (STZ, i.p. at 40 mg/kg) during the third week on the HFD in order to induce hyperglycemia by partial β-cell loss to establish the type 2 diabetes model.

Animals were stratified according to fasting blood glucose (244 ± 4 mg/dL) and body composition (25–26% body fat) as determined by nuclear magnetic resonance (NMR) spectroscopy, which was assessed after three weeks post-STZ injection. The mice were divided into six groups after a total of six weeks on the pre-study HFD (i.e. three weeks after STZ injection), and treated for ten weeks with isocaloric diets (4.16 ± 0.02 kcal/g) (n = 9–10) containing HAM-RS2. Sitagliptin was given at 0.4% weight of diet while HAM-RS2 was tested at three dosages: 0, 15, and 28% weight of diet. Prior to consumption by the rodents, powdered diets were formed into bars by mixing with water and freezing overnight in a -20°C freezer, allowing food to be placed in hoppers as opposed to cups or tins. Fasted BG and body composition were assessed at the end of the tenth week of treatment.

In the second study, male GLP-1R KO mice were used. The mice were treated with sitagliptin and HAM-RS2 (28% weight of diet) (SG+RS). The KO mice were obtained from Dr. Drucker at the University of Toronto, Ontario, Canada [10]. WT and KO mice (n = 12/group; N = 48) were generated through in-house breeding. Hyperglycemia was induced in the mice using the HFD + STZ protocol as described above. Baseline body weight (WT at 25 and KO at 28) was used to select the mice before HFD feeding, and blood glucose (220 mg/dL ± 3) was used for qualification of diabetic mice. The mice were then fed either a control diet or diet supplemented with SG+RS for eleven weeks.

### Energy balance

Metabolic rate and food intake were assessed by OxyMax/Comprehensive Lab Animal Monitoring System (Columbus Instruments International, Columbus, OH) for three days following a three-day acclimation period during the seventh and eighth weeks of the experimental. The chamber was maintained at the same temperature range and light-dark cycle as for the shoebox cages. Body composition and fasted BG were assessed at the end of ninth week and at the beginning of the tenth week of treatment.

### Blood glucose

Fasting blood glucose (BG) was tested using a glucometer (Abbott Laboratories, Inc., Chicago, IL). After overnight fasting, blood was drawn by either tail or submandibular bleeding using sterile 5 mm Goldenrod Animal Lancets (MEDI point, Inc., Mineola, NY).

### GLP-1, insulin, and leptin test

In the first study, plasma was used to determine the concentration of active GLP-1 using ALPCO Diagnostic’s GLP-1 ELISA (Active 7–36) kit (cat # 43-GP1HU-E01, Salem, NH). An EMD Millipore Multiplex MAP kit (cat# MMHMAG-44K St. Louis, MO) was used to assess the concentrations of various hormones including active GLP-1, leptin, and insulin in the second study. The GLP-1 assay used in the first study required plasma, which was collected in tubes containing another DPP-4 inhibitor (Millipore) after thirty minutes after re-feeding following an overnight fast. Fasting plasma was used for GLP-1 and other hormone tests in the second study.

### Tissue collection

Mice were sacrificed via decapitation and blood samples were collected in EDTA tubes. The blood was centrifuged at 4000g for 20 minutes in plasma extraction. Epididymal, perirenal, and retroperitoneal fat pads from the abdominal cavity were collected for total abdominal/visceral fat.

### Statistical Analysis

Data from the first and second studies were analyzed as 2x3 and 2x2 factorials, respectfully, using the MIXED procedure of SAS Version 9.3 (SAS Institute, Inc., Car, NC). Exceptions include pre-intervention data from the first study used to plan the second study, which were evaluated using student t-tests in Excel. The MIXED procedure was used to assure equal variance, normal distribution, and to identify outliers. Any observations that were more than three standard deviations away from the mean were considered outliers. Four total data points were removed: 28R1 and 28R5 were removed from GLP-1A and fasting BG measurements from the first study; KO11 and WTC9 from the second study’s insulin and leptin measurements, respectively. WTR/S1 and WTR/S2 were removed from food intake data due to suspected equipment malfunction. Data were transformed by log if the normality assumption was not met. This was followed by F-protected least significant difference mean comparison tests in order to determine differences among dietary treatments. Results were considered significant at p<0.05 and expressed as means ± standard error.

## Results

### Sitagliptin reduced adiposity in mice

**Fig 1:**
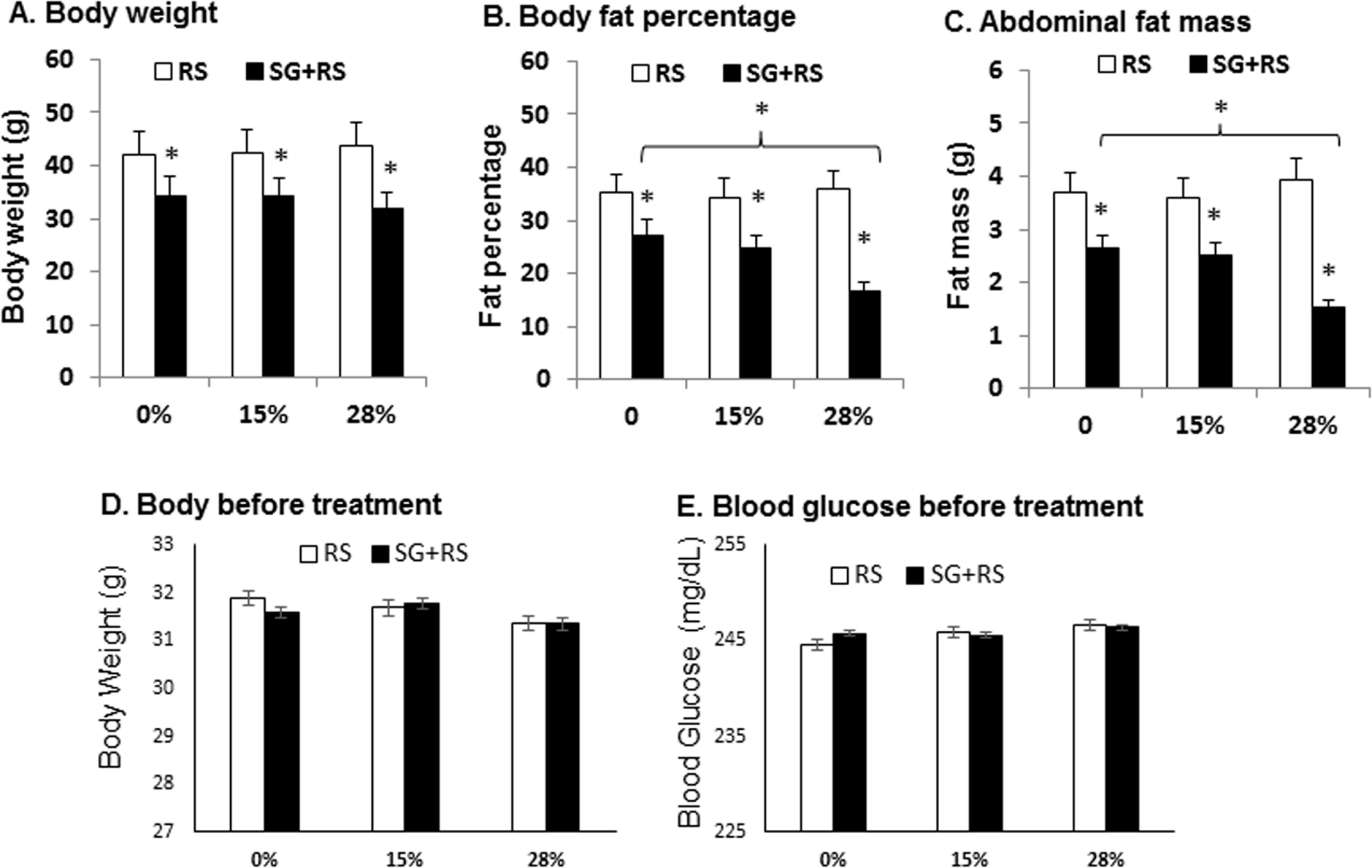
Body weight and adiposity. (A) Body weight of animals in response to treatment by SG and RS. (B) Body fat content as percentage of total weight. (C) Abdominal fat mass. (D) Body weight before treatment. (E) Blood glucose before treatment by SG and RG. The results are expressed as mean ± SEM (n = 9–10). * p<0.05 versus RS alone.

Sitagliptin was tested in the regulation of adiposity in type 2 diabetes mice, which was generated by a single injection of STZ into diet-induced obese mice. This obese and diabetic model was used in this study to mimic human type 2 diabetes, which is often associated with obesity. Pancreatic islets were partially impaired in the models by STZ to increase blood glucose further in the presence of insulin resistance in DIO mice. It is known that hyperglycemia is not strong in DIO mice. In this mouse model, sitagliptin reduced body weight, body fat content, and visceral fat (Fig 1A–1C). The effect was enhanced by resistant starch at 28% dosage (Fig 1B and 1C). Resistant starch alone did not exhibit an activity in the reduction of body weight, body fat, and visceral fat (Fig 1A–1C). There was no significant difference in body weight and blood glucose between the untreated and treated groups (Fig 1D and 1E). Blood glucose was above 240 mg/dl for qualification of diabetes in mice injected with STZ in this study. Blood glucose is below 100 mg/dl in normal lean mice. The inactivity of resistant starch is likely a result of fermentation inhibition by HFD (data not shown). The data suggest that sitagliptin is able to reduce adiposity and the effect is enhanced by resistant starch.

### Effect of sitagliptin on GLP-1R KO mice

**Fig 2:**
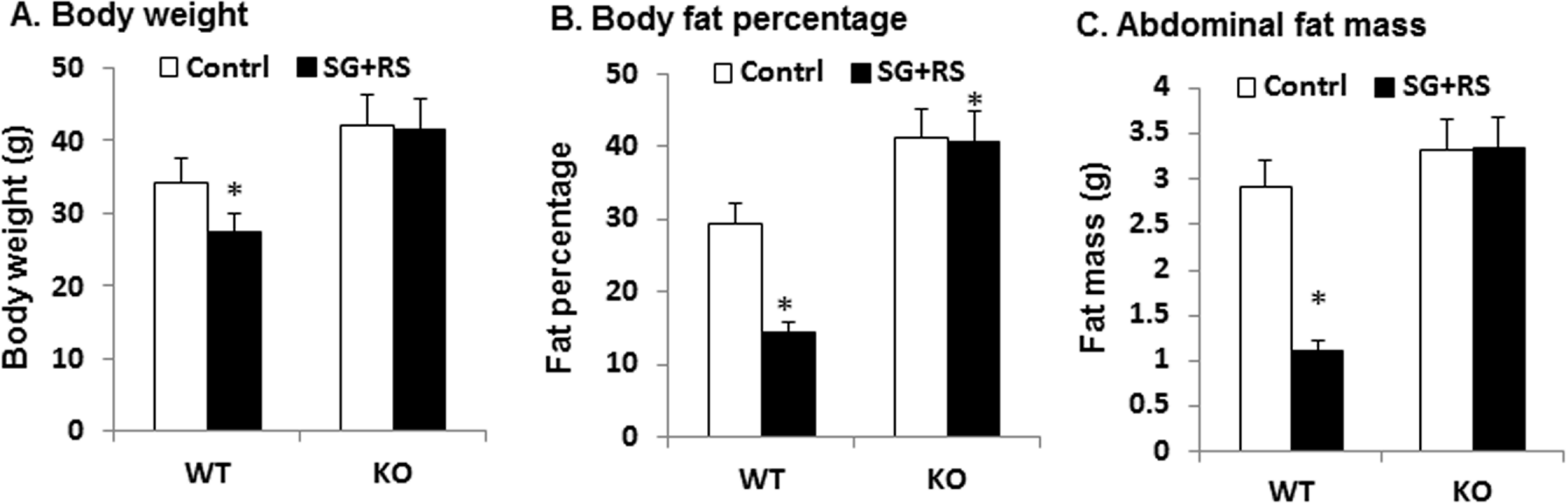
Body weight and adiposity in KO mice. A. Body weight of animals in response to SG and RS. B. Body fat content in percentage. C. Abdominal fat mass. The results are expressed as mean ± SEM (n = 12). * p<0.05 treated versus control.

To determine the role of GLP-1 in the effect of treatment, GLP-1 receptor knockout (KO) mice were used in this study. The GLP-1R gene was inactivated in KO mice as described elsewhere [10]. The mice were subject to treatment of sitagliptin in combination with 28% RS (SG+RS). The treatment decreased body weight, body fat, and visceral fat in WT mice (Fig 2A–2C). However, the responses were not observed in KO mice (Fig 2A–2C). These data demonstrate that the combined treatment reduced adiposity in WT mice, but not in KO mice.

### Sitagliptin induced energy expenditure

**Fig 3:**
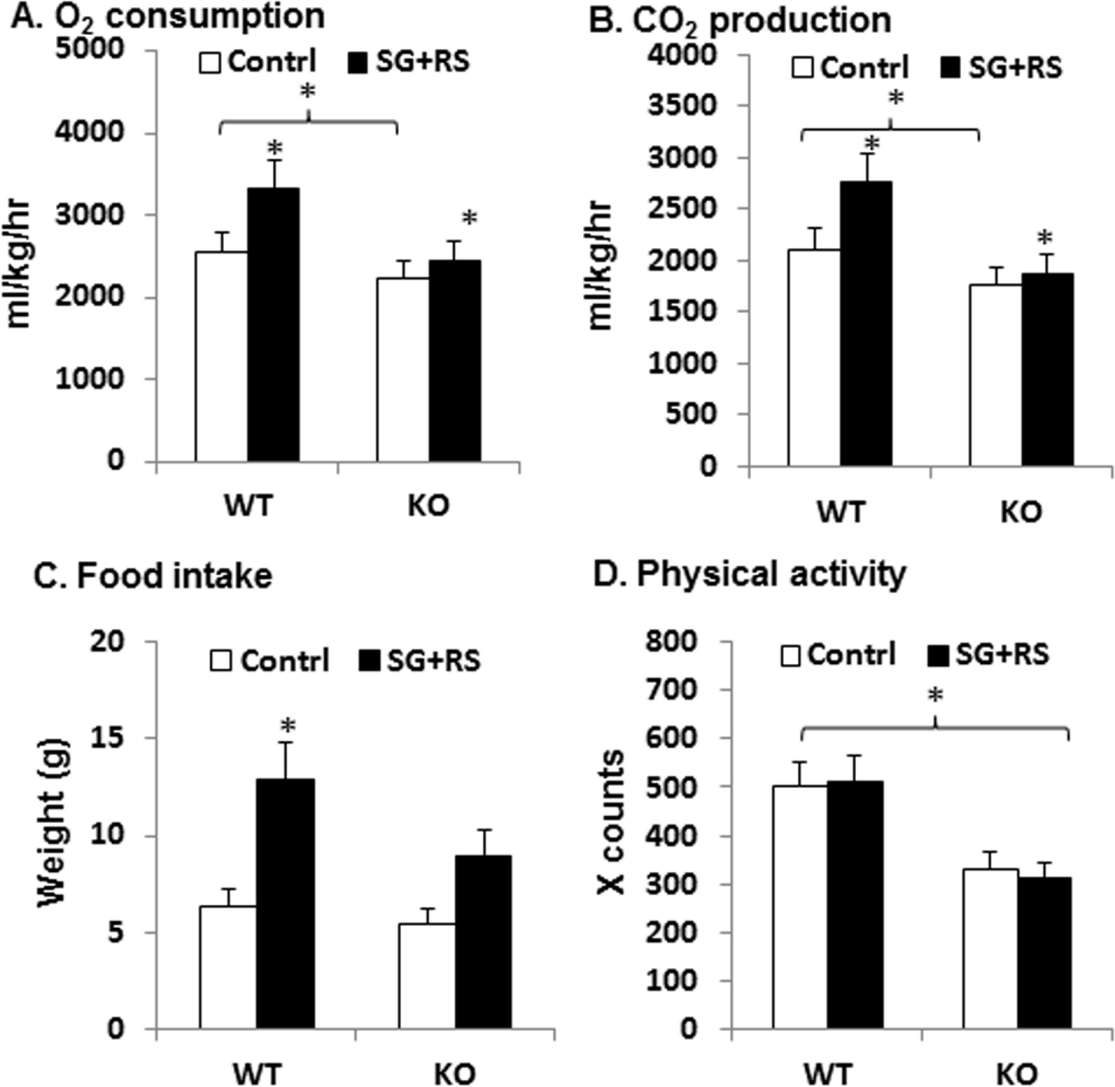
Energy expenditure. A. Oxygen consumption. B. Carbon dioxide production. C. Physical activity. D. Food intake. The results are expressed as mean ± SEM (n = 8). The results are expressed as mean ± SEM (n = 8). * p<0.05 treated vs. control or WT vs. KO.

Reduction in adiposity is usually a result of change in energy balance. Energy balance was assessed in mice using metabolic chambers. The treatment increased energy expenditure in mice as suggested by the increased oxygen consumption and carbon dioxide production (Fig 3A and 3B). The responses were observed in WT mice, but not in KO mice. Food intake was enhanced by the treatment in WT, but the increase was not significant in KO mice (Fig 3C). KO mice exhibited less physical activity than WT mice (Fig 3D), which is consistent with the lower energy expenditure as indicated by oxygen consumption and carbon dioxide production (Fig 3A and 3B). The treatment did not change the physical activity in WT or KO mice. This group of data demonstrates that SG+RS treatment enhanced energy expenditure, and food intake in WT mice, but not in KO mice. The data suggests that GLP-1 receptor is required for the effect of SG-RS treatment.

### Glucose metabolism in GLP-1R KO mice

**Fig 4:**
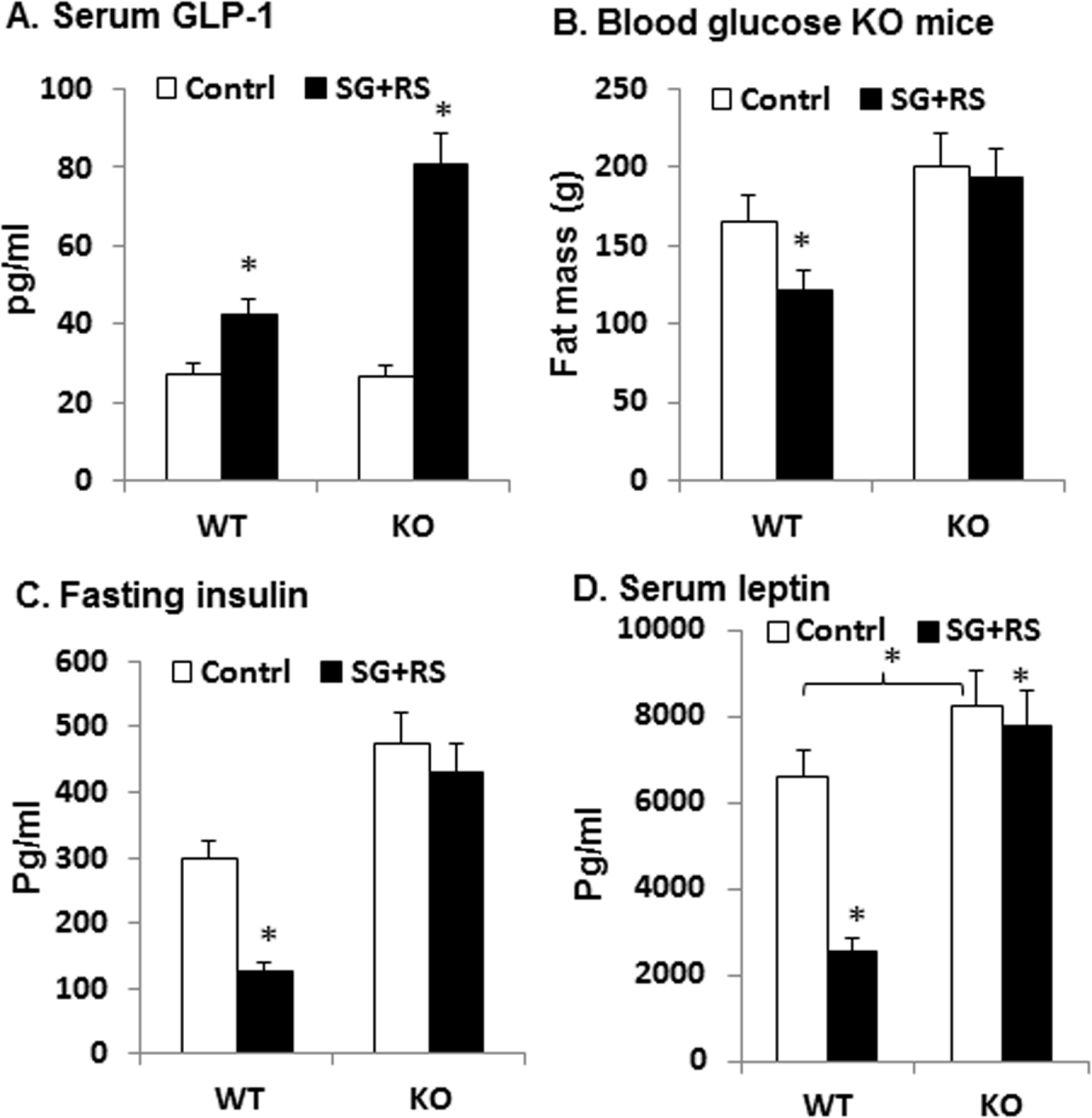
Serum GLP-1. A. Serum GLP-1 in mice. C. Fasting blood glucose. D. Fasting insulin. E. Serum leptin. The results are expressed as mean ± SEM (n = 12). * p<0.05 treated vs. control.

GLP-1 was examined in blood using the Multiplex kit to understand the mechanism of sitagliptin action. Serum GLP-1 was increased by 50% in WT mice by the treatment (Fig 4A). However, the increase was 200% in KO mice (Fig 4A). The increased GLP-1 was associated with a decrease in fasting glucose, fasting insulin, and serum leptin in WT mice (Fig 4B–4D). However, the association was not observed in KO mice even with the super elevation in GLP-1 (Fig 4B–4D). The data demonstrate that the treatment is able to improve glucose metabolism in WT mice, but not in KO mice. The result suggests that the effect in glucose metabolism is dependent on GLP-1R, and clearance of plasma GLP-1 is dependent on the receptor.

## Discussion

Our study suggests that administration of sitagliptin through dietary supplementation inhibits adiposity. It was reported in several studies that DPP-4 inhibitors, such as sitagliptin, had no effect on body weight [11–13]. In our study, body weight was reduced with body fat by sitagliptin. This observation is surprising to us. The weight reduction was observed in two cohorts of studies, and was associated with the increased energy expenditure. The increased energy expenditure was observed with sitagliptin plus resistant starch. The effect is likely from sitagliptin as sitagliptin alone reduced body weight in our model as shown in Fig 1. In contrast, resistant starch alone did not change body weight in our model. The weight loss and energy expenditure might be a result of a high dosage of sitagliptin due to dietary supplementation that was not observed in other studies.

The increased plasma GLP-1 is a potential mechanism of the weight loss. GLP-1 stimulates fat oxidation in both lean and obese conditions [14]. In rats, GLP-1 injection (i.v. at 50 pmol to 20 nmol) elicited dose-dependent increase in energy expenditure as indicated by oxygen consumption, heart rate, and core body temperature [15]. GLP-1 elevation after DPP-4 gene inactivation also reduces adiposity and weight gain in dietary obese mouse models [16, 17]. The effect on energy metabolism is likely dependent on GLP-1 activation of its receptor. Activation of GLP-1R by agonists reduces body weight in many studies [18–20]. Our study suggests that GLP-1 effect on weight reduction is dependent on GLP-1 receptor. The sitagliptin effect disappeared in GLP-1R KO mice. STZ may induce weight loss through inhibition of β-cell function. The weight loss is usually observed in a large dose of STZ. In this study, STZ was used only once at a very small dosage. The STZ treatment did not alter the weight gain rate in our DIO mice. Therefore, sitagliptin-induced weight loss may not be limited to our model system.

Our data suggest that GLP-1R may regulate serum level of GLP-1. The circulating GLP-1 concentration reflects a balance of secretion and degradation. The secretion by L-cells is induced by food intake. In this study, sitagliptin induced a greater elevation of GLP-1 in KO mice. GLP-1 secretion is induced by cAMP and ERK1/2 signaling pathways in L-cells, and such as reaction is induced by GLP-1 through activation of the receptor [21]. However, this possibility may not apply to the super induction of GLP-1 by sitagliptin in our KO mice. It is generally believed that a hormone receptor is required for clearance of the hormone in vivo. The super induction of GLP-1 in the receptor KO mice suggests that the principle may apply to GLP-1 clearance. The receptor deficiency may slow down GLP-1 clearance, leading to more accumulation of plasma GLP-1 in response to sitagliptin.

Food intake was increased by the treatment of sitagliptin plus resistant starch in this study via an unknown mechanism. One possibility is that the increased energy expenditure from the treatment makes the mice eat more in compensation for the increased calorie demand. Alternatively, the increased may be related to energy dilution by resistant starch in the diet although the diets were prepared to be isocaloric.

In summary, we observed that sitagliptin administration through dietary supplementation reduced adiposity and improved glucose metabolism in obese and diabetic mice. Sitagliptin activity in the inhibition of adiposity was enhanced by resistant starch at 28% of diet weight. The combined treatment enhanced energy expenditure in mice, which was associated with GLP-1 elevation. The effects of combined treatment are dependent on GLP-1 as the effects were lost in GLP-R1 KO mice. The activity of sitagliptin was enhanced by resistant starch.
